# Supplementary material for: Retention of volunteers and factors influencing program performance of the Senior Care Volunteers Training Program in Jiangsu, China
Source: PLoS One. 2020 Aug 10;15(8):e0237390. doi: 10.1371/journal.pone.0237390 (PMC7416946; doi:10.1371/journal.pone.0237390)
Supplement: S1 File — (DOC) [file pone.0237390.s001.doc]

**Evaluation of** **Senior Care Volunteers Training Program of Red Cross Society**

Dear the trained volunteer group leaders,

Red Cross Society of Jiangsu province has carried out the old-age care knowledge popularization and volunteer service work throughout the province since 2017. It obtained the great support from all levels of Red Cross Society in Jiangsu province and volunteers. School of Nursing, Nanjing medical university was entrusted to carry out the Senior Care Volunteers Training Program by Red Cross Society of Jiangsu. It gained a high level of affirmation and recognition from Red Cross Society of China and senior care volunteer teams of Red Cross Society. In order to systematically evaluate the implementation of training and provide reference for consolidate training in the next step, the survey was specially organized.

This survey is only for tracking and evaluating the training program performance, not for other purposes. Please fill it out according to the actual situation, so that we can improve the deficiencies. Thank you very much!

(After you complete this questionnaire, you will have a chance to get a *WeChat* cash red envelope.)

Senior care volunteer training program team of Red Cross Society of Jiangsu province

December 27, 2019

**Part 1, Survey on the demographic of trained volunteer group leader**

1. When did you attend the Senior Care Volunteer Training Program?

£2017 □2018 □2019

2. Which session did you attend?

□1st □2nd □3rd □4th

3. Date of birth： _______year _______month

4. Gender： £Female £Male

5. Marital status： □ Married £Others

6. Health status： £Excellent £Well £General £Poor

7. Whether care for the elderly at home： £Yes £No

8. Whether translate into parenthood： £Yes £No

9. Education： £Below high school £High school £Bachelor £Above bachelor

10. Employment： £Employed £Retired

11. Position： □ Doctors £Nurses £Preventive medicine workers

□ Social workers £Others

12. Work unit： £Nursing home £Medical institution £Others：_____________

13. Income of each month： □<2500 CNY £2500~3499 CNY £3500~4999 CNY

□≥5000 CNY

**Part 2 Survey on the information about volunteer team**

1. What is the full name of your senior care volunteer team?

2. Where is your volunteer team located?

City:

3. When did your senior care volunteer team start to carry out the volunteer activities for the elderly?

Year: Month:

4. How many activities have been carried out by your senior care volunteer team?

□<20 times £20-39 times □40-59 times □60-79 times

□80-99 times □≥100 times

5. How long is each activity on average in your volunteer team?

□<1 hour £1-2 hours □2-3 hours □3-4 hours □> 4 hours

6. How many volunteers in your volunteer team? (Including managers)

□<15 persons £15-29 persons □30-44 persons □45-59 persons □60-74 persons □75-89 persons □≥90 persons

7. What kinds of care for the senior are involved in your volunteer team? (Multiple choices)

□Safe care □Diet care □Drug manage care □Excretion care

□Clean care □Sleep care □Rehabilitation Care □Frailty care

□Chronic disease care □Psychological care □Others

9. If the annual finance was sufficient to support the voluntary service in your volunteer team?

□Inadequate □Adequate

**Part 3 Volunteer role identity**

Answer categories ranged from 1 to 7. 1 point indicates “strongly disagree”; 7 points indicates “strongly agree”. The higher the score, the stronger the degree.

1. My volunteer work is something I rarely ever think about.

| 1 Strongly disagree | 2 | 3 | 4 | 5 | 6 | 7 Strongly agree |
| --- | --- | --- | --- | --- | --- | --- |

2. I would feel a loss if I were forced to give up volunteering.

| 1Strongly disagree | 2 | 3 | 4 | 5 | 6 | 7 Strongly agree |
| --- | --- | --- | --- | --- | --- | --- |

3. I really don’t have any clear feelings about volunteer work.

| 1 Strongly disagree | 2 | 3 | 4 | 5 | 6 | 7Strongly agree |
| --- | --- | --- | --- | --- | --- | --- |

4. For me, being a volunteer means more than just doing volunteer work.

| 1 Strongly disagree | 2 | 3 | 4 | 5 | 6 | 7 Strongly agree |
| --- | --- | --- | --- | --- | --- | --- |

5. Volunteering is an important part of who I am.

| 1 Strongly disagree | 2 | 3 | 4 | 5 | 6 | 7 Strongly agree |
| --- | --- | --- | --- | --- | --- | --- |

**Part 4 Attitude toward helping others**

Answer categories ranged from 1 to 5. 1 point indicates “strongly disagree”; 5 points indicates “strongly agree”. The higher the score, the stronger the degree.

1. I think people should be willing to help others who are less fortunate.

| 1 Strongly disagree | 2 | 3 | 4 | 5 Strongly agree |
| --- | --- | --- | --- | --- |

2. Helping troubled people with their problems is very important to me.

| 1 Strongly disagree | 2 | 3 | 4 | 5 Strongly agree |
| --- | --- | --- | --- | --- |

3. I think people should be more charitable toward others in society.

| 1 Strongly disagree | 2 | 3 | 4 | 5 Strongly agree |
| --- | --- | --- | --- | --- |

4. I think people in need should receive support from others.

| 1 Strongly disagree | 2 | 3 | 4 | 5 Strongly agree |
| --- | --- | --- | --- | --- |

**Part 5 Team climate and atmosphere**

Answer categories ranged from 1 to 5. 1 point indicates “strongly disagree”; 5 points indicates “strongly agree”. The higher the score, the stronger the degree.

1. Team members trust each other in my volunteer team.

| 1 Strongly disagree | 2 | 3 | 4 | 5 Strongly agree |
| --- | --- | --- | --- | --- |

2. Morale on my volunteer team is high.

| 1 Strongly disagree | 2 | 3 | 4 | 5 Strongly agree |
| --- | --- | --- | --- | --- |

3. Team members support each other in my volunteer team.

| 1 Strongly disagree | 2 | 3 | 4 | 5 Strongly agree |
| --- | --- | --- | --- | --- |

4. There are no feelings among team members which might pull my volunteer team apart.

| 1 Strongly disagree | 2 | 3 | 4 | 5 Strongly agree |
| --- | --- | --- | --- | --- |

5. My volunteer team resolves conflicts soon after they occur.

| 1 Strongly disagree | 2 | 3 | 4 | 5 Strongly agree |
| --- | --- | --- | --- | --- |

6. I feel free to express my opinions in my volunteer team.

| 1 Strongly disagree | 2 | 3 | 4 | 5 Strongly agree |
| --- | --- | --- | --- | --- |

7. I have an influence on team decisions.

| 1 Strongly disagree | 2 | 3 | 4 | 5 Strongly agree |
| --- | --- | --- | --- | --- |

8. Team members can openly discuss their own problems and issues in my volunteer team.

| 1 Strongly disagree | 2 | 3 | 4 | 5 Strongly agree |
| --- | --- | --- | --- | --- |

9. Team members show consideration for needs and feelings of other team members in my volunteer team.

| 1 Strongly disagree | 2 | 3 | 4 | 5 Strongly agree |
| --- | --- | --- | --- | --- |

10. Team members receive recognition for individual performance in my volunteer team.

| 1 Strongly disagree | 2 | 3 | 4 | 5 Strongly agree |
| --- | --- | --- | --- | --- |

**Part 6 Volunteer Program Performance Evaluation**

Answer categories ranged from 0 to 10, with 0 indicating "absolutely not" and 10 indicating "absolutely yes". The higher the score, the stronger the degree (the meaning of "yes" and "no" is determined according to the specific connotation of each item).

**Relevance**

1. How **responsive** is your volunteer team to the needs of the elderly?

| 0 No response | 1 | 2 | 3 | 4 | 5 | 6 | 7 | 8 | 9 | 10 Prompt response |
| --- | --- | --- | --- | --- | --- | --- | --- | --- | --- | --- |

2. How **urgent** is the volunteer service provided by your team for the development of senior care in your area?

| 0 Not urgent | 1 | 2 | 3 | 4 | 5 | 6 | 7 | 8 | 9 | 10 Very urgent |
| --- | --- | --- | --- | --- | --- | --- | --- | --- | --- | --- |

3. Whether the senior care provided by your team is **a priority care program** in the local communities / families / nursing homes?

| 0 Absolutely not | 1 | 2 | 3 | 4 | 5 | 6 | 7 | 8 | 9 | 10 Absolutely yes |
| --- | --- | --- | --- | --- | --- | --- | --- | --- | --- | --- |

4. Whether specific volunteer activities provided by your team are **suitable for** the elderly?

| 0 Absolutely unsuitable | 1 | 2 | 3 | 4 | 5 | 6 | 7 | 8 | 9 | 10 Absolutely suitable |
| --- | --- | --- | --- | --- | --- | --- | --- | --- | --- | --- |

5. Whether the senior care provided by your team is consistent with the **purpose** of your team?

| 0 Absolutely inconsistent | 1 | 2 | 3 | 4 | 5 | 6 | 7 | 8 | 9 | 10 Absolutely consistent |
| --- | --- | --- | --- | --- | --- | --- | --- | --- | --- | --- |

6. Whether the senior care provided by your team is consistent with the **functions** of your team?

| 0 Absolutely inconsistent | 1 | 2 | 3 | 4 | 5 | 6 | 7 | 8 | 9 | 10 Absolutely consistent |
| --- | --- | --- | --- | --- | --- | --- | --- | --- | --- | --- |

7. Whether the volunteers’ **overall behavior** is appropriate when implementing the volunteer care for the elderly?

| 0 Absolutely unsuitable | 1 | 2 | 3 | 4 | 5 | 6 | 7 | 8 | 9 | 10 Absolutely suitable |
| --- | --- | --- | --- | --- | --- | --- | --- | --- | --- | --- |

8. Whether the volunteers’ **overall communication methods** are appropriate when implementing the volunteer care for the elderly?

| 0 Absolutely unsuitable | 1 | 2 | 3 | 4 | 5 | 6 | 7 | 8 | 9 | 10 Absolutely suitable |
| --- | --- | --- | --- | --- | --- | --- | --- | --- | --- | --- |

9. Whether the volunteers’ **attitudes** are appropriate when implementing the volunteer care for the elderly?

| 0 Absolutely unsuitable | 1 | 2 | 3 | 4 | 5 | 6 | 7 | 8 | 9 | 10 Absolutely suitable |
| --- | --- | --- | --- | --- | --- | --- | --- | --- | --- | --- |

**Efficiency**

1. How **cost-effective** is your volunteer team?

| 0 Very bad | 1 | 2 | 3 | 4 | 5 | 6 | 7 | 8 | 9 | 10 Very good |
| --- | --- | --- | --- | --- | --- | --- | --- | --- | --- | --- |

2. How **complete** is an activity in your team in a given period of time?

| 0 Unfinished | 1 | 2 | 3 | 4 | 5 | 6 | 7 | 8 | 9 | 10 All finished |
| --- | --- | --- | --- | --- | --- | --- | --- | --- | --- | --- |

3. How well is the senior care in your team **promoted locally**?

| 0 No promotion | 1 | 2 | 3 | 4 | 5 | 6 | 7 | 8 | 9 | 10 Very wide |
| --- | --- | --- | --- | --- | --- | --- | --- | --- | --- | --- |

4. How well is the senior care in your team **promoted in other regions**?

| 0 No promotion | 1 | 2 | 3 | 4 | 5 | 6 | 7 | 8 | 9 | 10 Very wide |
| --- | --- | --- | --- | --- | --- | --- | --- | --- | --- | --- |

**Effectiveness**

1. Does the content of the senior care provided by your team **follow the original plan**?

| 0 Not follow | 1 | 2 | 3 | 4 | 5 | 6 | 7 | 8 | 9 | 10 Exactly follow |
| --- | --- | --- | --- | --- | --- | --- | --- | --- | --- | --- |

2. How well are the activities of the project completed as planned in your volunteer team each year?

| 0 Unfinished | 1 | 2 | 3 | 4 | 5 | 6 | 7 | 8 | 9 | 10 All finished |
| --- | --- | --- | --- | --- | --- | --- | --- | --- | --- | --- |

3. To what extent has the program's **positive impact on aging attitudes** of the elderly who received services changed?

(Aging attitude refers the process of aging and the evaluation and experience of aging, including positive and negative. It has an important impact on people's behavior, self-concept, acceptance of aging, lifestyle, etc., and also affect the elderly mental health.)

| 0 Not change | 1 | 2 | 3 | 4 | 5 | 6 | 7 | 8 | 9 | 10 Change very large |
| --- | --- | --- | --- | --- | --- | --- | --- | --- | --- | --- |

4. Whether the **expectation of aging** of the elderly who received services has improved?

(The expectation of aging refers the expectation level of the elderly to achieve and maintain physical and mental functions, and it is the concrete expression of the health beliefs of the elderly on the problem of aging.)

| 0 Not improve | 1 | 2 | 3 | 4 | 5 | 6 | 7 | 8 | 9 | 10 Improve very much |
| --- | --- | --- | --- | --- | --- | --- | --- | --- | --- | --- |

5. To what extent has **the elderly’s care-giving knowledge** improved?

| 0 Not improve | 1 | 2 | 3 | 4 | 5 | 6 | 7 | 8 | 9 | 10 Improve very much |
| --- | --- | --- | --- | --- | --- | --- | --- | --- | --- | --- |

6. To what extent have **the elderly’s care-giving skills** improved?

| 0 Not improve | 1 | 2 | 3 | 4 | 5 | 6 | 7 | 8 | 9 | 10 Improve very much |
| --- | --- | --- | --- | --- | --- | --- | --- | --- | --- | --- |

7. To what extent has **relevant persons’ (family members, care staffs of elderly families / nursing homes) care-giving knowledge** improved?

| 0 Not improve | 1 | 2 | 3 | 4 | 5 | 6 | 7 | 8 | 9 | 10 Improve very much |
| --- | --- | --- | --- | --- | --- | --- | --- | --- | --- | --- |

8. To what extent have **relevant persons’ (family members, care staffs of elderly families / nursing homes) care-giving skills** improved?

| 0 Not improve | 1 | 2 | 3 | 4 | 5 | 6 | 7 | 8 | 9 | 10 Improve very much |
| --- | --- | --- | --- | --- | --- | --- | --- | --- | --- | --- |

**Satisfaction**

1. How satisfied are **the elderly** who received volunteer care services with **the content of the services**?

| 0 Dissatisfied | 1 | 2 | 3 | 4 | 5 | 6 | 7 | 8 | 9 | 10 Satisfied |
| --- | --- | --- | --- | --- | --- | --- | --- | --- | --- | --- |

2. How satisfied are **the elderly** who received volunteer care services with **the time chosen for the services**?

| 0 Dissatisfied | 1 | 2 | 3 | 4 | 5 | 6 | 7 | 8 | 9 | 10 Satisfied |
| --- | --- | --- | --- | --- | --- | --- | --- | --- | --- | --- |

3. How satisfied are **the elderly** who received volunteer care services with **volunteer service attitudes**?

| 0 Dissatisfied | 1 | 2 | 3 | 4 | 5 | 6 | 7 | 8 | 9 | 10 Satisfied |
| --- | --- | --- | --- | --- | --- | --- | --- | --- | --- | --- |

4. How satisfied are **the elderly** who received volunteer care services with **the outcome of the service program**?

| 0 Dissatisfied | 1 | 2 | 3 | 4 | 5 | 6 | 7 | 8 | 9 | 10 Satisfied |
| --- | --- | --- | --- | --- | --- | --- | --- | --- | --- | --- |

5. How satisfied are **the elderly families /nursing homes** receiving volunteer care services with **the content of the services**?

| 0 Dissatisfied | 1 | 2 | 3 | 4 | 5 | 6 | 7 | 8 | 9 | 10 Satisfied |
| --- | --- | --- | --- | --- | --- | --- | --- | --- | --- | --- |

6. How satisfied are **the elderly families /nursing homes** receiving volunteer care services with **the time chosen for the services**?

| 0 Dissatisfied | 1 | 2 | 3 | 4 | 5 | 6 | 7 | 8 | 9 | 10 Satisfied |
| --- | --- | --- | --- | --- | --- | --- | --- | --- | --- | --- |

7. How satisfied are **the elderly families /nursing homes** receiving volunteer care services with **volunteer service attitudes**?

| 0 Dissatisfied | 1 | 2 | 3 | 4 | 5 | 6 | 7 | 8 | 9 | 10 Satisfied |
| --- | --- | --- | --- | --- | --- | --- | --- | --- | --- | --- |

8. How satisfied are **the elderly families /nursing homes** receiving volunteer care services with **the outcome of the service program**?

| 0 Dissatisfied | 1 | 2 | 3 | 4 | 5 | 6 | 7 | 8 | 9 | 10 Satisfied |
| --- | --- | --- | --- | --- | --- | --- | --- | --- | --- | --- |

**Impact**

1. Is there any change in the **choice of elderly care methods** for the elderly who received senior care services because of the content of the project?

| 0 Not change | 1 | 2 | 3 | 4 | 5 | 6 | 7 | 8 | 9 | 10 Change very large |
| --- | --- | --- | --- | --- | --- | --- | --- | --- | --- | --- |

2. To what extend have the elderly families and nursing homes receiving services **accepted** the senior care services?

| 0 Totally unacceptable | 1 | 2 | 3 | 4 | 5 | 6 | 7 | 8 | 9 | 10 Fully accept |
| --- | --- | --- | --- | --- | --- | --- | --- | --- | --- | --- |

3. To what extend has your **volunteer team’s building capacity** improved?

| 0 Not improve | 1 | 2 | 3 | 4 | 5 | 6 | 7 | 8 | 9 | 10 Improve very much |
| --- | --- | --- | --- | --- | --- | --- | --- | --- | --- | --- |

4. To what extend have **volunteers’ abilities** in your volunteer team improved? (Such as communication skill, organizational management skill, value judgment skill, etc.)

| 0 Not improve | 1 | 2 | 3 | 4 | 5 | 6 | 7 | 8 | 9 | 10 Improve very much |
| --- | --- | --- | --- | --- | --- | --- | --- | --- | --- | --- |

5. Are **the media** concerned about your team's the senior care program activities?

| 0 Not be concerned | 1 | 2 | 3 | 4 | 5 | 6 | 7 | 8 | 9 | 10 Be concerned a lot |
| --- | --- | --- | --- | --- | --- | --- | --- | --- | --- | --- |

6. How well is **finance support** from government and social projects?

| 0 Not support | 1 | 2 | 3 | 4 | 5 | 6 | 7 | 8 | 9 | 10 Support a lot |
| --- | --- | --- | --- | --- | --- | --- | --- | --- | --- | --- |

7. To what extent has the senior care provided by your team improved the **social elderly's ideology of respect for the elderly**?

| 0 Not improve | 1 | 2 | 3 | 4 | 5 | 6 | 7 | 8 | 9 | 10 Improve very much |
| --- | --- | --- | --- | --- | --- | --- | --- | --- | --- | --- |

8. Whether there is any **guidance** provided by other agencies?

| 0 No guidance | 1 | 2 | 3 | 4 | 5 | 6 | 7 | 8 | 9 | 10 A lot of guidance |
| --- | --- | --- | --- | --- | --- | --- | --- | --- | --- | --- |

**Sustainability**

1. Whether the **risk assessment system** is **perfect** in your volunteer team?

| 0 Imperfect | 1 | 2 | 3 | 4 | 5 | 6 | 7 | 8 | 9 | 10 Perfect |
| --- | --- | --- | --- | --- | --- | --- | --- | --- | --- | --- |

2. Whether the **event plans** are **complete** in your volunteer team?

| 0 Uncomplete | 1 | 2 | 3 | 4 | 5 | 6 | 7 | 8 | 9 | 10 Complete |
| --- | --- | --- | --- | --- | --- | --- | --- | --- | --- | --- |

3. Whether the **activities** are **feasible** in our volunteer team?

| 0 No | 1 | 2 | 3 | 4 | 5 | 6 | 7 | 8 | 9 | 10 Yes |
| --- | --- | --- | --- | --- | --- | --- | --- | --- | --- | --- |

4. Are the elderly who received services **willing to continue receiving the services** of this project in the future?

| 0 Unwilling | 1 | 2 | 3 | 4 | 5 | 6 | 7 | 8 | 9 | 10 Willing |
| --- | --- | --- | --- | --- | --- | --- | --- | --- | --- | --- |

5. Whether **the team management system** is **standard** in your volunteer team?

| 0 Unstandardized | 1 | 2 | 3 | 4 | 5 | 6 | 7 | 8 | 9 | 10 Standardized |
| --- | --- | --- | --- | --- | --- | --- | --- | --- | --- | --- |

6. How **stable are the team members** in your volunteer team?

| 0 Unstable | 1 | 2 | 3 | 4 | 5 | 6 | 7 | 8 | 9 | 10 Stable |
| --- | --- | --- | --- | --- | --- | --- | --- | --- | --- | --- |

7. Whether the **volunteer protection system** is **complete** in your volunteer team?

| 0 Uncomplete | 1 | 2 | 3 | 4 | 5 | 6 | 7 | 8 | 9 | 10 Complete |
| --- | --- | --- | --- | --- | --- | --- | --- | --- | --- | --- |

8. Can volunteers master **the knowledge** of senior care?

| 0 No | 1 | 2 | 3 | 4 | 5 | 6 | 7 | 8 | 9 | 10 Yes |
| --- | --- | --- | --- | --- | --- | --- | --- | --- | --- | --- |

Overall self-assessment: Are the above questions filled out carefully?

□Yes □No

Thank you again for your cooperation!

It is with people like you who are willing to actively contribute to the senior care of Red Cross Society cause that the society and the senior care are getting better and better! Let's fight side by side and keep doing!
